# Coagulopathy and the humoral response against viral proteins in patients at different stages of COVID-19

**DOI:** 10.1590/0074-02760220072

**Published:** 2023-01-20

**Authors:** Fernanda Pereira Monteiro, Victor de Souza Tavares, Rayane da Silva Oliveira Souza, Larissa Paola Rodrigues Venâncio, Mary Hellen Fabres-Klein, Rodrigo Feliciano do Carmo, Raphael Contelli Klein, Jonilson Berlink Lima, Théo Araújo-Santos

**Affiliations:** 1 Universidade Federal do Oeste da Bahia, Centro das Ciências Biológicas e da Saúde, Núcleo de Estudos de Agentes Infecciosos e Vetores, Barreiras, BA, Brasil; 2 Universidade Federal do Vale do São Francisco, Colegiado de Ciências Farmacêuticas, Petrolina, PE, Brasil

**Keywords:** D-dimer, coagulopathy, immunoglobulin humoral response, SARS-CoV-2, COVID-19

## Abstract

**BACKGROUND:**

Patients with severe coronavirus disease 2019 (COVID-19) often present with coagulopathies and have high titres of circulating antibodies against viral proteins.

**OBJECTIVES:**

Herein, we evaluated the association between D-dimer and circulating immunoglobulin levels against viral proteins in patients at different clinical stages of COVID-19.

**METHODS:**

For this, we performed a cross-sectional study involving patients of the first wave of COVID-19 clinically classified as oligosymptomatic (n = 22), severe (n = 30), cured (n = 27) and non-infected (n = 9). Next, we measured in the plasma samples the total and fraction of immunoglobulins against the nucleoprotein (NP) and the receptor-binding domain (RBD) of the spike proteins by enzyme-linked immunosorbent assay (ELISA) assays.

**FINDINGS:**

Patients with severe disease had a coagulation disorder with high levels of D-dimer as well as circulating IgG against the NP but not the RBD compared to other groups of patients. In addition, high levels of D-dimer and IgG against the NP and RBD were associated with disease severity among the patients in this study.

**MAIN CONCLUSIONS:**

Our data suggest that IgG against NP and RBD participates in the worsening of COVID-19. Although the humoral response against severe acute respiratory syndrome coronavirus 2 (SARS-CoV-2) is partially understood, and more efforts are needed to clarify gaps in the knowledge of this process.

The severe acute respiratory syndrome coronavirus 2 (SARS-CoV-2) is an enveloped virus with single-stranded RNA that is the etiological agent of coronavirus disease 2019 (COVID-19). SARS-CoV has four structural proteins, namely the spike (S), envelope (E), membrane (M) and nucleocapsid phosphoprotein (NP).^1^ The S protein is necessary for viral entry into host cells as it contains the receptor-binding domain (RBD).^2^ The RBD is part of the S1 subunit of the S protein and comprises the fragment that binds to the human angiotensin-converting enzyme 2 (ACE2) present on the cell surface.^2^ ACE2 plays a central role as the cellular receptor for viral entry into the cell. NP recovers the viral genomic RNA and impairs host effector mechanisms of viral RNA degradation.^3^ ^,^ ^4^

As viral entry into the human cell depends on the interaction between the RBD and ACE2, antibodies specifically targeting this domain have high neutralising activity against the virus. In contrast, antibodies against NP, cannot neutralise the virus.^5^ Previous studies on SARS-CoV found an association between high titres of anti-NP antibodies and poor outcomes in infected patients.^6^ ^,^ ^7^ However, no mechanism has been suggested to explain the relationship between severity and the humoral response against NP. In this context, antibodies against the RBD protein of SARS-CoV may protect the organism against infection, while antibodies targeted at other regions of the S protein may even enhance infection.^8^

A mechanism that may be associated with the pathophysiology of the disease is the onset of systemic coagulation, which involves the emergence of thrombotic events, such as deep vein thrombosis (DVT) and pulmonary embolism (PE), alongside arterial and systemic thrombosis or even during extracorporeal membrane oxygenation (ECMO) treatment in extracorporeal circuits. In this regard, it was shown that such patients have antibodies that can trigger a coagulation cascade like a haemophagocytic syndrome.^9^ ^,^ ^10^

Herein, we investigated the titres of immunoglobulins in patients with different clinical forms of COVID-19 as indicated by circulating D-dimer levels in the patient plasma. We also describe the production of specific IgGs against the nucleoprotein (NP) and the receptor binding domain (RBD) of the spike protein of SARS-CoV-2 in patients with different clinical forms of COVID-19.

## MATERIALS AND METHODS

*Ethics statement* - The Research Ethics Committee of UFOB approved this study in 2020 (license number: 30629520.6.0000.0008). All clinical investigations were conducted according to the Declaration of Helsinki.

*Study design* - This was a cross-sectional study of COVID-19 cases registered in cities in the western region of Bahia, Brazil, from May to October 2020. Available laboratory and clinical data from non-infected health professionals (n = 9) and patients with oligosymptomatic (n = 22) and severe (n = 30) disease at the Hospital do Oeste were collected. The exclusion criterion for infected patients was the absence of a confirmation of the disease by quantitative polymerase chain reaction (qPCR). Clinical data were collected from patients in the cured group (n = 27) through interviews; additionally, blood samples were collected from patients. Then, an immune enzymatic assay was performed to detect different isoforms of immunoglobulins as described below.

*Viral load determination* - Swab samples obtained from patients reporting COVID-19-like symptoms were processed for SARS-CoV-2 detection via quantitative real-time PCR (RT-qPCR) using the USCDC protocol. In brief, sample RNA was extracted using commercial kits, such as the PureLink^®^ Viral RNA/DNA Mini Kit (ThermoScientific), Cellco (Cellco Biotec) and Biogene (Quibasa) according to the supplier’s instructions; the RNA was then resuspended in 60 µL of RNase-free water (GIBCO). Next, RT-qPCR was performed according to the manufacturer’s instructions with the QuantStudio 5 Real-Time PCR system (ThermoScientific, USA) using the primer set kits (IDT Coralville, IA) and KAPA PROBE FAST qPCR Master Mix (2X) Kit (Sigma-Aldrich). Then, viral load was determined using dilutions of a plasmid containing the SARS-CoV-2 N gene to produce a standard curve (IDT Coralville, IA).

*Enzyme-linked immunosorbent assay (ELISA)* - Total immunoglobulins and fractions thereof against the SARS-CoV-2 NP and RBD were measured in plasma samples from COVID-19 patients. Briefly, 96-well plates were sensitised with 50 μL of a solution containing 2 μg/mL of the RBD or NP and stored at 4ºC overnight; thereafter, the supernatant was discarded, and the plates were washed four times with 200 μL of PBS-T. Non-specific binding was blocked by incubating the plates with 200 μL of blocking solution (PBS-T with 3% milk) for 2 h at room temperature (RT). Then, the supernatants were discarded. Next, 50 μL of patient samples diluted 1:10 in PBS-T with 1% milk was added per well and the plates were incubated at 4ºC overnight. The supernatants were discarded, and the plates were washed four times with 200 μL of PBS-T. Then, the samples were incubated with 50 μL of peroxidase-conjugated specific secondary antibody diluted in PBS-T plus 1% milk and incubated for 1 h at RT. The dilution factor was 1:5000 for anti-IgA, 1:5000 for anti-IgGAM, 1:3000 for anti-IgG Fab, 1:1500 for anti-IgG Fc, 1:3000 for anti-IgG3 or anti-IgG4. Next, the supernatants were discharged, and the plates were washed four times with 200 µL of PBS-T. For development, 100 μL of OPD was added per well and incubated for 30 min at RT. Then, 50 μL of 3 M HCl was added to terminate the enzymatic reaction. Readings were obtained using a multimode microplate reader at wavelengths of 492 and 650 nm (Varioskan Lux, Thermo Fischer Scientific). The cut off was established by means of the mean plus twice the standard deviation value optical density (OD) of the uninfected samples for each immunoglobulin tested.

*Rapid tests for antibodies against SARS-CoV-2* - All samples were tested for the presence of SARS-CoV-2 antibodies using commercial immunochromatographic tests according to the manufacturer’s instructions (ECO Diagnostica, Nova Lima, MG).

*D-dimer measurement* - Circulating D-dimer levels were measured in plasma from COVID-19 patients using a commercial ELISA kit according to the manufacturer’s instructions (Thermo Fisher Scientific).

*Statistical analysis* - Statistical analyses were performed using GraphPad-Prism 5.0 software (GraphPad Software, San Diego, CA, USA). All data are represented as the median (IQR) or n (%). All data were statistically analysed using the Kruskal-Wallis test and Dunn’s post-test, χ² test or Mann-Whitney test. The contingency data were analysed using Fisher’s exact test. p < 0.05 was considered statistically significant. All experiments were performed with at least three repetitions.

## RESULTS

This study enrolled 88 patients who were classified by their clinical and laboratory features into groups of non-infected (n = 9), oligosymptomatic (n = 22), severe (n = 30), and cured (n = 27) individuals. A total of thirteen patients in the study population died. According to previously obtained data, patients with severe disease had a higher average age (66 years, p < 0.0001, Kruskal-Wallis test) compared with the other groups.^11^ ^,^ ^12^ ^,^ ^13^ The gender distribution did not differ significantly between groups. We also quantified COVID-19 markers in our study population. We did not find statistically significant differences in the viral load between the oligosymptomatic and severe groups. However, patients with severe disease had higher titres of D-dimer. Additionally, the frequency of diabetes and hypertension was higher in the severe group compared with the other groups. Regarding the symptoms evaluated, only the frequency of myalgia and dyspnoea differed among the groups. While myalgia was more common among oligosymptomatic patients, dyspnoea was more frequent in patients with severe disease (Table).

**TABLE:** Clinical characteristics of coronavirus disease 2019 (COVID-19) patients

Characteristic	Non-infected (n = 9)	Oligosymptomatic (n = 22)	Severe (n = 30)	Cured (n = 27)	p-value
Demographic data					
Age (years)	32 (± 1.765)	33 (± 1.977)	66 (± 3.099)	38 (± 2.177)	< 0.0001^a^
Female, sex (n%)	7 (78%)	14 (64%)	19 (63%)	15 (56%)	0.6885^b^
COVID-19 marker					
D-Dimer (mg/L)	-	0.5274 (± 0.013)	0.6073 (± 0.011)	0.4368 (± 0.013)	< 0.0001^a^
Viral charge (GC/mL)	-	10740000 (± 53750000)	2033000 (± 251100000)	-	0.0727^c^
Comorbidities					
Asthma	1 (11%)	1 (5%)	0 (0%)	1 (4%)	-
Diabetes	0 (0%)	0 (0%)	7 (23%)	1 (4%)	-
Hypertension	0 (0%)	0 (0%)	10 (33%)	2 (7%)	-
Symptoms					
Myalgia	1 (11%)	12 (55%)	2 (7%)	3 (11%)	< 0.0001^b^
Edema	0 (0%)	1 (5%)	0 (0%)	3 (11%)	-
Dyspnoea	1 (11%)	2 (9%)	16 (53%)	6 (22%)	0.0018^b^
Loss of smell / taste	0 (0%)	8 (36%)	2 (7%)	5 (19%)	-
Diarrhoea	0 (0%)	5 (23%)	1 (3%)	3 (11%)	-
Vomiting	0 (0%)	3 (14%)	1 (3%)	1 (4%)	-

All data are presented as the median (IQR) or n (%). All data are calculated applying a Kruskal-Wallis test (a), χ² test (b) or Mann Whitney test (c). p < 0.05 was considered statistically significant.

**Fig. 1:**
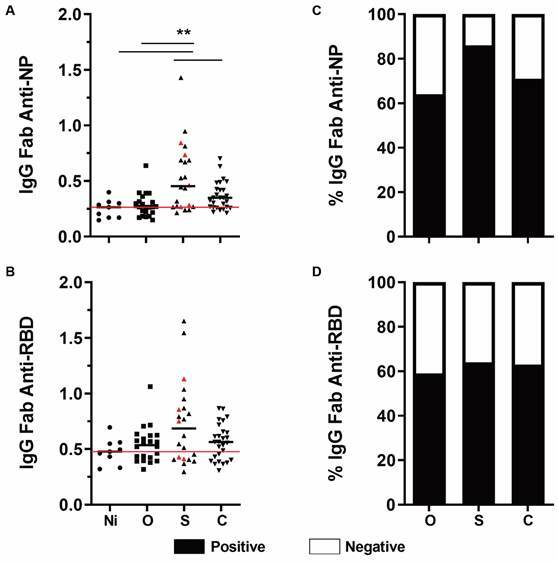
plasma levels of IgG Fab against the severe acute respiratory syndrome coronavirus 2 (SARS-CoV-2) nucleoprotein (NP) and receptor-binding domain (RBD) in patients at different clinical disease stages. Plasma levels of IgG Fab were measured in non-infected (NI, n = 9), oligosymptomatic (O, n = 21), severe (S, n = 21) or cured (C, n = 27) coronavirus disease 2019 (COVID-19) patients. The panels of the graphs represent the optical density (OD) (492-650 nm) of IgG Fab against (A) NP and (B) RBD. The left panels indicate the contingency of positive data in each patient group against (C) NP and (D) RBD. Red dots represent patient deaths, and the red line indicates the median of the NI group. Data were analysed using the Kruskal-Wallis test and Dunn’s post-test. The p-values are indicated in each graph by **(p < 0.01).

**Fig. 2:**
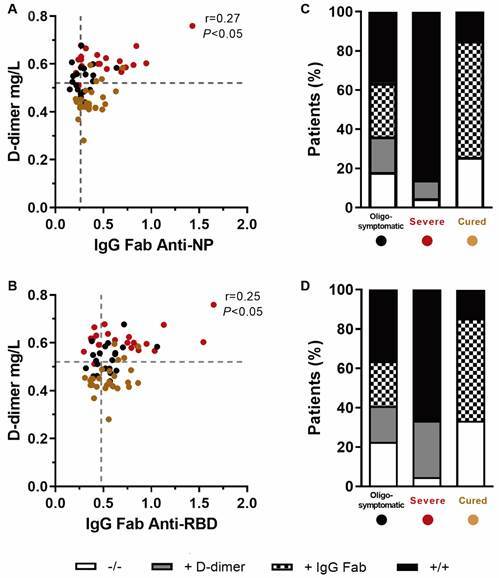
coagulopathy and the IgG response against specific viral proteins in patients at different stages of coronavirus disease 2019 (COVID-19). D-dimer and plasma levels of immunoglobulins against the nucleoprotein (NP) and receptor-binding domain (RBD) of spike protein-I were measured in patients with oligosymptomatic (O, n = 20), severe (S, n = 21) or cured (C, n = 27) COVID-19. Data on the graphs represent correlations between D-dimer and the (A) anti-NP or (B) anti-RBD IgG Fab. The dotted lines in the X and Y axes indicate the median optical density (OD) value of the negative group (492 - 650 nm) and D-dimer cut-off (0.52 mg/L), respectively. (C-D) The contingency of positive data in each patient group, separated according to the result obtained for D-dimer and IgG Fab levels: white = doubly negative; grey = positive for D-dimer; checkered = positive for IgG Fab, and black = double positive. Data were analysed using Spearman’s test. The R and p*-*values are shown in each graph.

To identify differences in the humoral response against the RBD and NP of SARS-CoV-2 between healthy controls and the oligosymptomatic, severe and cured groups, we screened samples for the presence of several immunoglobulin subclasses [Supplementary data (Figs 1-2)]. The accuracy of the ELISA method used for the evaluation was confirmed by comparing the IgG titres of severely infected patients with the results of the rapid antibody tests. Our results showed that most patients tested had higher OD values than the median values of non-infected patients, including patients considered negative by the rapid test, indicating greater sensitivity of the ELISA compared to the rapid test [Supplementary data (Fig. 3)]. We observed high plasma IgG levels against the NP in the severe group compared to the cured group (Fig. 1A), but no difference in anti-RBD antibody levels was observed between the groups (Fig. 1B). Moreover, the contingency data showed that NP was present in more than 80% of the severely infected patients (Fig. 1C-D).

D-dimer values have been shown to increase during the aggravation of the disease (Table).^14^ ^,^ ^15^ ^,^ ^16^ Herein, we verified the correlation between immunoglobulin titres and D-dimer levels in the plasma of individuals at different clinical stages of COVID-19 [Supplementary data (Figs 4-5)]. Interestingly, only IgG-Fab showed a significant correlation with NP (p < 0.05, Spearman test; Fig. 2A) and RBD antigens (p < 0.05, Spearman test; Fig. 2B). To identify a specific severity signature, we stratified patients using cut-offs for anti-RBD and anti-NP IgG and D-dimer levels (Fig. 2C-D). Severely infected patients had increased levels of both IgG and D-dimer compared to the other groups, with 18/21 (85%) to anti-NP IgG and 14/21 (65%) to anti-RBD IgG. However, the relationship between antibodies against SARS-CoV-2 proteins and coagulation remains to be determined.

## DISCUSSION

Neutralising antibodies are associated with protection against SARS-CoV-2 and other viruses. In the context of COVID-19, the humoral response seems to be transient, since antibody levels decrease over time.^17^ In addition, several studies have shown a correlation between high levels of immunoglobulins and disease severity.^17^ ^,^ ^18^ In this study, we observed high levels of circulating IgG against the NP and RBD of SARS-CoV-2, suggesting that the humoral response can be related to the pathophysiology of COVID-19.

Since early in the pandemic, scientists have identified high levels of circulating antibodies in convalescent patients.^5^ ^,^ ^19^ ^,^ ^20^ In addition, patients with severe disease display more populations of B cells compared with oligosymptomatic patients.^21^ ^,^ ^22^ ^,^ ^23^ However, clinical trials that tested plasma derived from COVID-19 patients failed to protect treated patients from death.^24^ ^,^ ^25^ Herein, we observed low immunoglobulin levels in oligosymptomatic patients. These patients did not test positive for antibodies via commercial rapid antibody tests but displayed high viral loads as tested by reverse transcription polymerase chain reaction (RT-PCR) until Day 18 of symptoms. These data suggest a delay in the development of a humoral response against the structural proteins of SARS-CoV-2. Other study have indicated that antibodies failed to distinguish asymptomatic COVID-19 patients.^26^ These data suggest that the humoral response can be related to poor disease prognosis.

Although the relationship between antibody production and severe disease seems clear,^27^ the association between coagulopathy and the humoral response has not been addressed thus far. Severe COVID-19 is characterised by systemic thrombosis, pulmonary venous congestion and embolism.^28^ ^,^ ^29^ In addition, several studies have identified D-dimer and coagulation factors as biomarkers of severe disease.^30^ ^,^ ^31^ ^,^ ^32^ Herein, we identified high levels of circulating D-dimer associated with anti-NP and anti-RBD IgG only in severely infected patients. However, we cannot infer any mechanistic effect of circulating antibody levels on thrombotic events in COVID-19.

This study has some limitations since a small number of patients was investigated. In addition, the patients were enrolled during the first wave of the disease when only the Wuhan variant of the virus has been detected; new variants of SARS-CoV-2 were identified in Brazil in November 2020.^33^ These new variants are associated with other symptoms, although coagulopathy remains the major symptom related to mortality.

Altogether, our data suggest an association between D-dimer levels and the humoral response against RBD and NP viral proteins in patients with severe COVID-19. Further studies should be conducted to clarify the causal role of antibodies in the coagulation disturbances associated with the disease. However, the study of the human humoral response to the first contact with SARS-COV-2 is only possible by analysing individuals affected by COVID-19 during the first wave of the pandemic, which is one of the limitations for studies after the beginning of the vaccination period.
