# Supplementary material for: Coagulopathy and the humoral response against viral proteins in patients at different stages of COVID-19
Source: Mem Inst Oswaldo Cruz. 2023 Jan 20;117:e220072. doi: 10.1590/0074-02760220072 (PMC9870257; doi:10.1590/0074-02760220072)
Supplement: Supplementary file 1 [file 1678-8060-mioc-117-e220072-s.pdf]

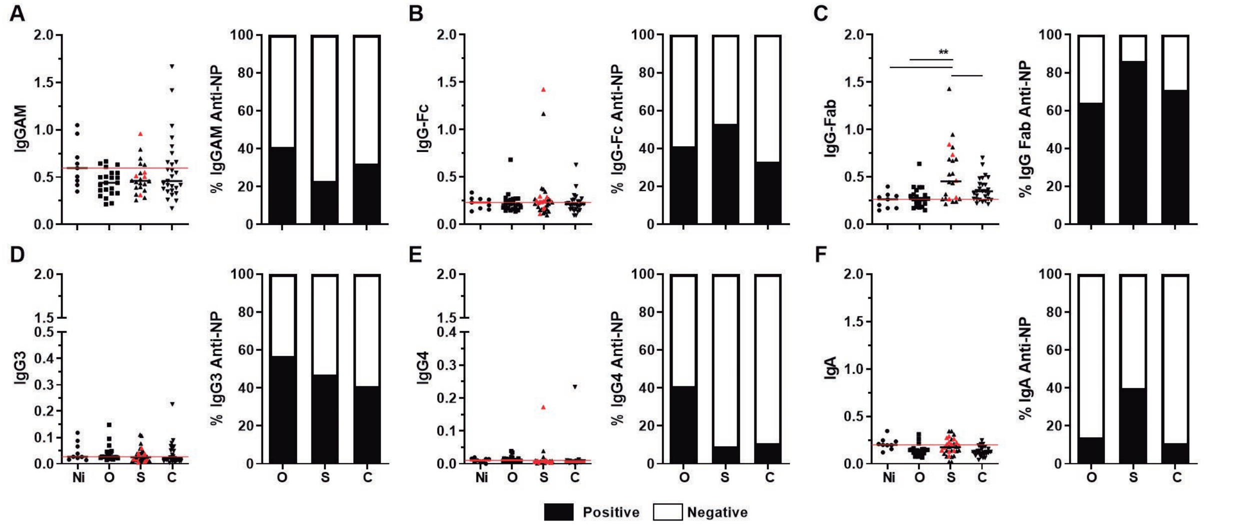


Fig. 1: total immunoglobulins and fractions thereof against the SARS-CoV-2 NP in patients in different clinical disease stages. Plasma levels of immunoglobulins were measured in non-infected (NI, n = 9), oligosymptomatic (O, n = 22), severe (S, n = 22 or 30) or cured (C, n = 27) COVID-19 patients. The right panels of the graph represent the OD (492-650 nm) of (A) IgGAM, (B) IgG Fc, (C) IgG Fab, (D), IgG3, (E) IgG4 and (F) IgA. The left panels indicate the contingency of positive data in each patient group. Red dots represent patient deaths, and the red line indicates the median of the NI group. Data were analysed using the Kruskal-Wallis test and Dunn's post-test. The p-values are indicated in each graph by **p < 0,01.

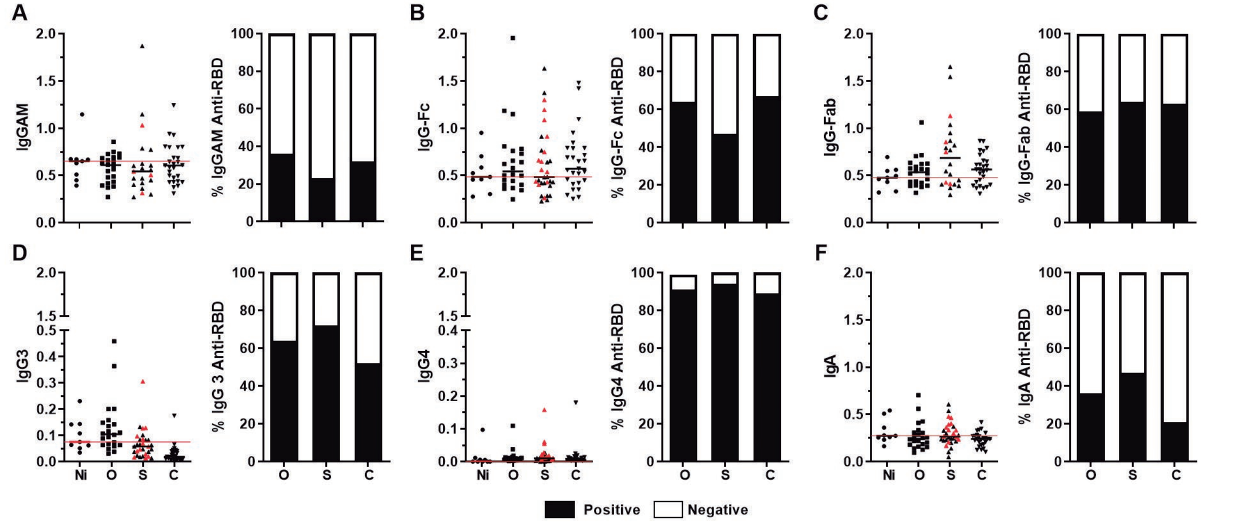


Fig. 2: total immunoglobulins and fractions thereof against the SARS-CoV-2 RBD in patients in different clinical disease stages. Plasma levels of immunoglobulins were measured in non-infected (NI, n = 9), oligosymptomatic (O, n = 22), severe (S, n = 22 or 30) or cured (C, n = 27) stage of COVID-19. Right panels of the graphs represent the OD (492-650 nm) of (A) IgGAM, (B) IgG Fc, (C) IgG Fab, (D), IgG3, (E) IgG4 and (F) IgA. The left panels indicate the contingency of positive data in each patient group. Red dots represent patient deaths, and the red line indicates the median of the NI group. Data were analysed using the Kruskal-Wallis test and Dunn's post-test.

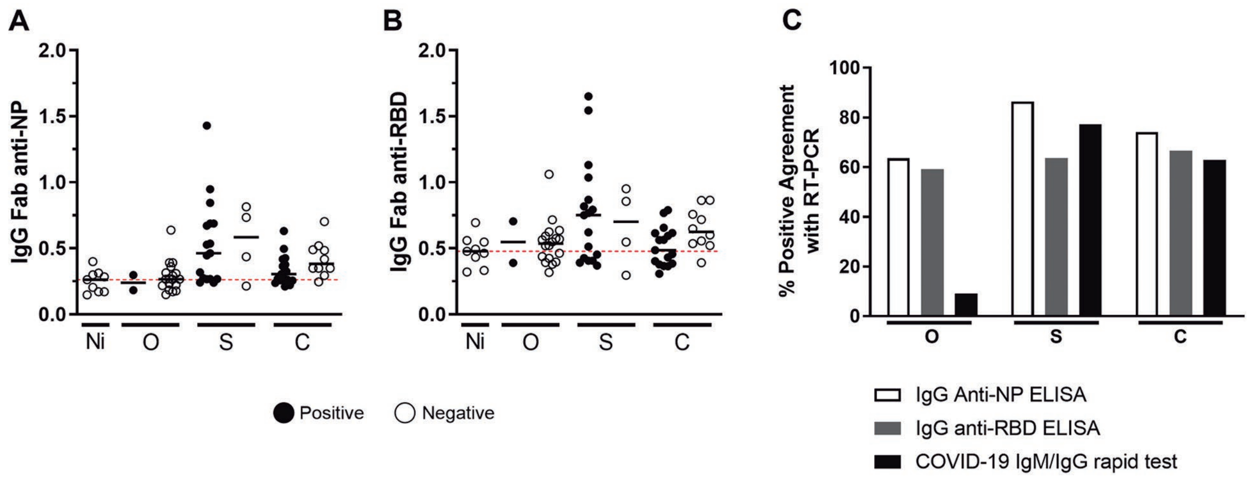


Fig. 3: validation of anti-RBD and Anti-NP IgG in house ELISA test using RT-PCR and a commercial test as reference. (A) anti-NP and (B) anti-RBD IgG Fab were measured in plasma samples of patients non-infected (NI, n = 9), oligosymptomatic (O, n = 20), severe (S, n = 21) or cured (C, n = 27) for COVID-19. All patients recruited were previously tested for SARS-CoV-2 infection by RT-qPCR test, except the cured patients. Next, plasma samples were tested using commercial antibody tests to COVID-19, which detects IgM and IgG antibodies. Graphs represent the OD (492-650 nm) of IgG Fab against (A) NP and (B) RBD proteins. Dots on the graphs indicates positives (filled circles) and negative (empty circles) samples by rapid tests. Red dashed line indicates the median of the NI group. (C) Bars on the graphs indicate the contingency of positive agreement with RT-PCR for the data in each patient group.

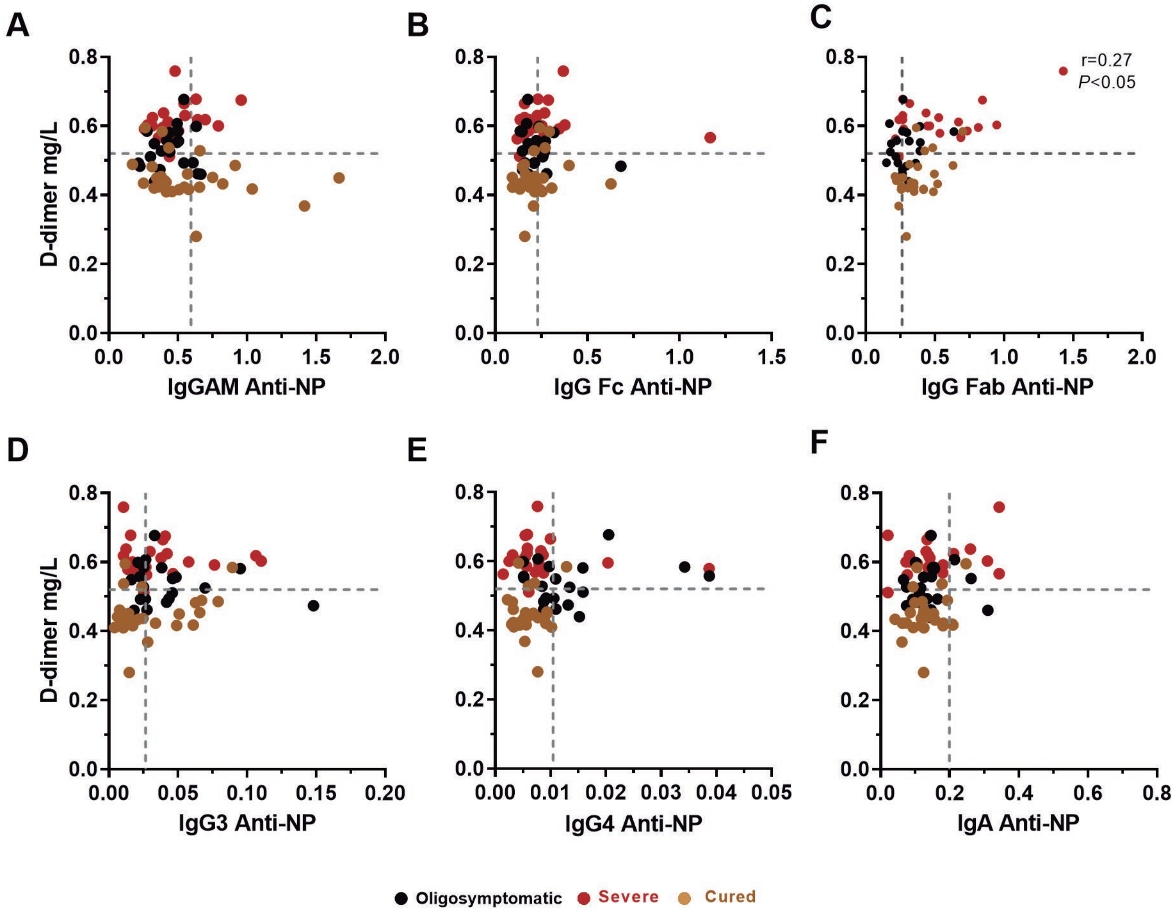


Fig. 4: correlation between D-dimers total and subclass immunoglobulins against SARS-COV-2 nucleocapsid protein. D-dimer and plasma levels of immunoglobulins against NP were measured in patients with oligosymptomatic (n = 20), severe (n = 21) or cured (n = 27) COVID-19. Data on the graphs represents correlations between D-dimer and (A) IgGAM, (B) IgG Fc, (C) IgG Fab, (D) IgG3, (E) IgG4, (F) IgA, against SARS-COV-2 RBD. The dotted lines in the X and Y axes indicate the median OD value of the negative group (492-650 nm) and D-dimer cut-off (0.52 mg/L), respectively. Data were analysed using Spearman's test. The R and p-values are shown in each graph.

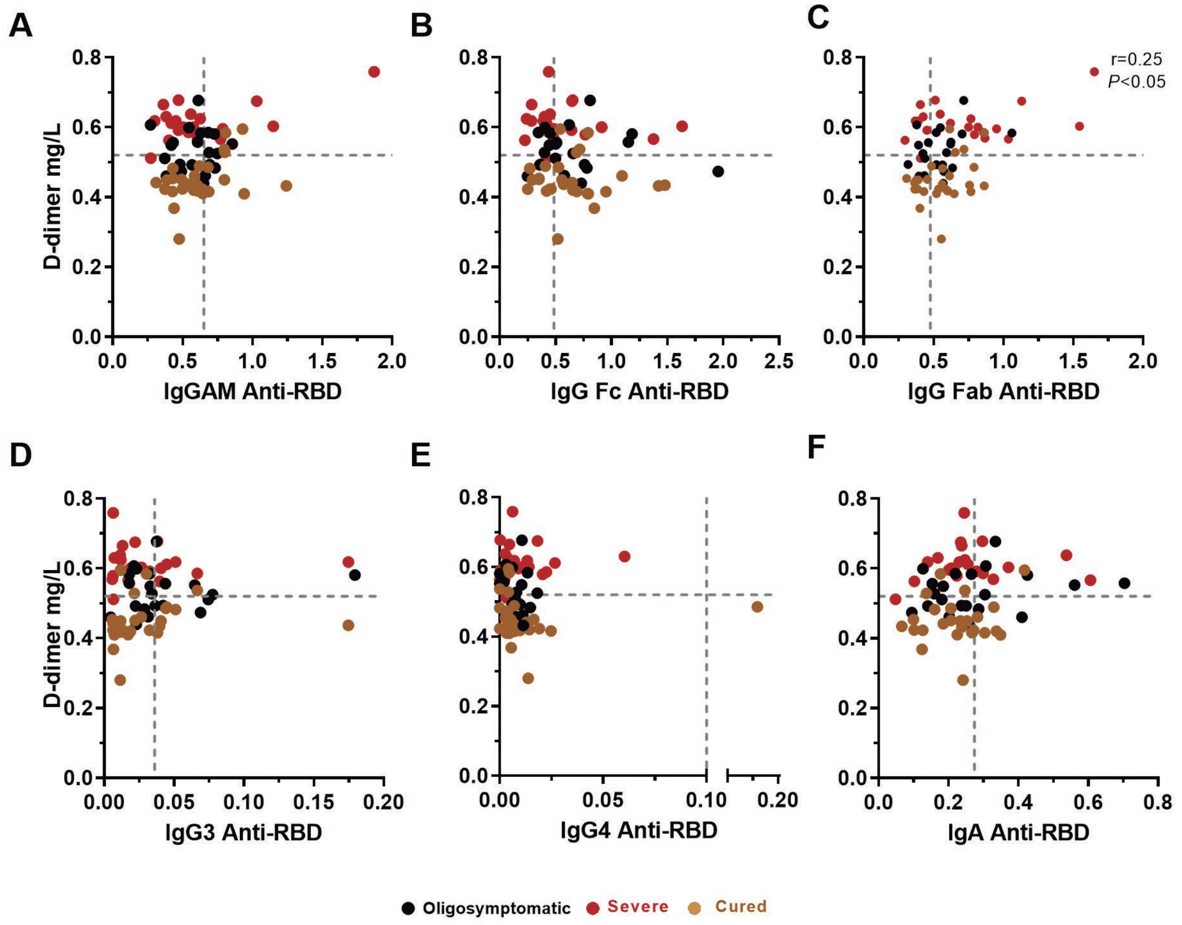


Fig. 5: correlation between D-dimers total and subclass immunoglobulins against SARS-COV-2 receptor bind domain. D-dimer and plasma levels of immunoglobulins against RBD of spike protein-I were measured in patients with oligosymptomatic (n = 20), severe (n = 21) or cured (n = 27) COVID-19. Data on the graphs represents correlations between D-dimer and (A) IgGAM, (B) IgG Fc, (C) IgG Fab, (D) IgG3, (E) IgG4, (F) IgA, against SARS-COV-2 RBD. The dotted lines in the X and Y axes indicate the median OD value of the negative group (492-650 nm) and D-dimer cut-off (0.52 mg/L), respectively. Data were analysed using Spearman's test. The R and p-values are shown in each graph.
